# Role of T_EFFECTOR/MEMORY_ Cells, *TBX21* Gene Expression and T-Cell Homing Receptor on Type 1 Reaction in Borderline Lepromatous Leprosy Patients

**DOI:** 10.1371/journal.pone.0164543

**Published:** 2016-10-20

**Authors:** Luciana Nahar dos Santos, Pedro Henrique Lopes da Silva, Iris Maria Peixoto Alvim, José Augusto da Costa Nery, Flávio Alves Lara, Euzenir Nunes Sarno, Danuza Esquenazi

**Affiliations:** 1 Leprosy Laboratory, Oswaldo Cruz Institute, Oswaldo Cruz Foundation, Rio de Janeiro, Brazil; 2 Laboratory of Cellular Microbiology, Oswaldo Cruz Institute, Oswaldo Cruz Foundation, Rio de Janeiro, Brazil; 3 Department of Pathology and Laboratories, School of Medical Sciences, State University of Rio de Janeiro, Rio de Janeiro, Brazil; Mie University Graduate School of Medicine, JAPAN

## Abstract

In spite of hyporesponsivity to *Mycobacterium leprae*, borderline lepromatous (BL) patients show clinical and immunological instability, and undergo frequent acute inflammatory episodes such as type 1 reaction (T1R), which may cause nerve damages. This work focused on the participation of T cell subsets from blood and skin at T1R onset. We observed a significantly increased *ex vivo* frequency of both effector and memory CD4+ and CD8+ T cells in T1R group. Besides, *ex vivo* frequency of T cell homing receptor, the Cutaneous Leukocyte-associated Antigen (CLA) was significantly increased in T cells from T1R patients. *M*. *leprae* induced a higher frequency of CD4+ T_EM_ and CD8+ T_EF_ cells, as well as of CD8+/TEMRA (terminally differentiated effector T cells) subset, which expressed high CD69+. The presence of IFN-γ‒producing-CD4+ T_EF_ and naïve and effector CD8+ T lymphocytes was significant in T1R. *TBX21* expression was significantly higher in T1R, while BL showed increased *GATA3* and *FOXP3* expression. In T1R, *TBX21* expression was strongly correlated with CD8+/IFN-γ‒ T cells frequency. The number of double positive CD8+/CLA+ and CD45RA+/CLA+ cells was significantly higher in skin lesions from T1R, in comparison with non-reactional BL group. The observed increase of *ex vivo* T cells at T1R onset suggests intravascular activation at the beginning of reactional episodes. The antigen-specific response in T1R group confirmed the higher number of CD8+/CLA+ and CD45RA+/CLA+ cells in T1R lesions suggests possible migration of these cells activated by *M*. *leprae* components inside the vascular compartment to skin and participation in T1R physiopathology.

## Introduction

Leprosy is a chronic infectious disease caused by the obligate intracellular pathogen *Mycobacterium leprae*, which affects about 200,000 new individuals worldwide [1]. *M*. *leprae* preferably infects skin macrophages and Schwann cells from peripheral nerves, and the variety of clinical and pathological features of the disease according to the host immune response gives rise to a spectrum of polar forms. At the lepromatous pole, patients showing anergy or hyporesponsivity to *M*. *leprae* antigens and present disseminated lesions with high bacillary load, as opposed to tuberculoid ones, who exhibit a preserved specific cellular immune response, with limited lesions and a restricted growth of the pathogen. The so-called borderline forms (BL, BB and BT) are intermediary and range between the two poles [2].

The major cause of deformities and neural disabilities in leprosy relates to immune reactions that affect 30–50% of patients during the clinical course of the disease. Reactional episodes are characterized by a sudden, intense and unregulated inflammatory response, being subdivided into Reversal Reaction (T1R or RR) and *Erythema Nodosum Leprosum* (T2R or ENL) [3, 4].

Although the triggering mechanisms of such reactions still require a better clarification, some studies describe risk factors that would be related to the development thereof, such as the bacillary load and the clinical forms. However, literature also suggests other factors, such as age, gender and the presence of co-infections, and several combinations between them may be related to the type of reaction under examination [4, 5].

T1R presents a gradual development, and its natural course may last several weeks. It primarily affects borderline patients, being rarely detected in polar lepromatous patients. As to its clinical aspects, T1R is characterized by an increased inflammatory process in pre-existing skin lesions, as well as by the appearance of new granulomatous lesions and localized set of symptoms [5]. In T1R patients, cell-mediated immune response is the predominant cause of neuritis, and, if not suitably treated with corticosteroids, it provokes disabilities and deformities. Indeed, T1R is the leading cause of physical impairment in leprosy [6].

Among borderline patients, immunopathology of T1R is still poorly understood and most studies do not discriminate borderline forms [7], [8] BL patients are clinically unstable and should be studied on a separate basis. While BT skin lesions show granuloma formation with a predominance of epithelioid and giant cells without *M*. *leprae*, BL patients present a diffuse inflammatory infiltrate, with a large number of T lymphocytes in the dermis and abundant bacilli. At the onset of T1R clinical symptoms, BL patients also develop granulomas in the skin lesions and produce mediators of the inflammatory response [9, 10], suggesting that T cells can be activated and play a critical role in the immunopathogenesis of these episodes.

The most recent studies on T-cell participation in leprosy only encompass the characterization of responses to different *M*. *leprae* antigens, almost always combined with sorologic tests, aiming at finding a biomarker of exposure to the pathogen and to the early diagnosis of the infection [11, 12]. Originally described by Sallusto et al., T-cell subsets are differentiated according to the expression of surface molecules [13]. Among them, one should particularly refer to CCR7 and CD45RA. Thus, T_NAÏVE_ cells present CCR7+/CD45RA+ phenotype, central memory (T_CM_) are CCR7+/CD45RA-, effector memory (T_EM_) are CCR7-/CD45RA-, and effector cells (T_EF_) are CCR7-/CD45RA+ [14, 15]. Several subsets of T-cells have been showing a relevant participation in the immunopathology of infectious diseases, including memory T-cells, which used to be well-known only by virtue of the protective role played by them [16, 17]. However, there is still a few number of studies on the effective participation of different T-cells subsets in the pathogenesis of leprosy *per se*, as well as on the reactional episodes thereof.

In this context, the main purpose of our work was to determine the participation of *ex vivo* and *in vitro* T-cell response to *M*. *leprae* in blood and skin lesions from BL patients at the onset of T1R. Indeed, evaluations of the T-cell phenotype with special attention to activation/homing, cytokine production and memory profile were performed as a possible contribution to understand the pathogenesis of T1R in this form of leprosy.

## Material and Methods

### Ethical considerations

The study was approved by the Institutional Ethics Committee of the Oswaldo Cruz Foundation/FIOCRUZ (permit protocol number 518/09) and an informed written consent was obtained from all individuals prior to specimen collection.

### Studied population

This study included 32 individuals, among whom 12 were BL patients with T1R (immediately after diagnosis of the reactional episode and without use of immunosuppressant drugs), 10 were non-reactional BL patients immediately after diagnosis and before the beginning of multidrugtherapy (MDT). All patients were diagnosed according to Ridley and Jopling [18] criteria and accompanied at Leprosy Outpatient Unit–FIOCRUZ. We also used blood samples from 10 healthy volunteers (HV) with the same social-economic background as the patients and living in Rio de Janeiro city, which is known to be endemic for leprosy. Neither leprosy patients below 15 years-old nor any other affected by acute or chronic infectious comorbidities were included in this study. For the sake of privacy and well-being of the studied individuals, we refrained from disclosing their identity.

### PBMC collection and culture and *in vitro* stimulation assays

Peripheral blood mononuclear cells (PBMC) were obtained under endotoxin free conditions from heparinized venous blood of patients and healthy donors in Ficoll-Hypaque (GE Healthcare, Uppsala, AB, Sweden) density centrifugation. After a separation, part of freshly isolated PBMC were resuspended at 1x10^6^/mL in PBS for *ex vivo* analysis and the remaining were cultured in AIM V (Gibco BRL, Gaithersburg, MD, USA) at 5 x 10^5^ of cultured PBMC/well for 6 hours in 96-well U bottom culture plates (Costar, Cambridge, MA, USA) in the presence of 1μg/mL anti-CD28 and anti-CD49d co-stimulatory molecules (BD Bioscience, San José, CA, USA). Then, the cells were stimulated with 1μg/mL of enteroxin B from *Staphylococcus aureus* (SEB, Sigma, St. Louis, MO, USA) or 20μg/mL of irradiated and sonicated armadillo-derived *M*. *leprae* (ML; supplied under the agreement NIH/NIAID contract N01 AI-25469 with Colorado State University, CO, USA). For the assays of intracellular cytokines detection, the cultures were kept at 37°C with 5% CO_2_ and 70% humidity, and, during the last two hours, 10 μg/mL of the protein transport inhibitor brefeldin A was added (GolgiPlug, BD Bioscience). The kinetics of responses to *M*. *leprae* and SEB were previously determined in healthy volunteers, reaching a peak at 6 hour-cultures.

### Analysis of surface molecules and intracellular cytokines on CD4+ and CD8+ T subsets by flow cytometry

Newly obtained PBMC (*ex vivo*) together with the 6-hour cultures were resuspended in PBS (Gibco) 0,02% ethylenediamine tetraacetic acid (EDTA; Sigma) and stained with DAPI (Live/Dead Kit, Invitrogen, Grand Island, NY, USA) for separation of dead cells, according to the manufacturer’s instructions. Briefly, PBMC were incubated with DAPI (Invitrogen) for 30 minutes, washed by centrifugation and once more incubated in PBS containing Fc-receptor blocking solution (Biolegend Inc., San Diego, CA, USA). After a new wash step, PBMC were resuspended in flow cytometry buffer (PBS with 1% FCS and 0.01% sodium azide, all from Gibco) and incubated for 30 minutes at 4°C, with surface monoclonal antibodies anti-CD3 V500, anti-CCR7 PerCp, anti-CD4 or anti-CD8 APC, anti-CD69 APC-Cy7, anti-CD45RA Alexa Fluor 488 and anti-CLA FITC (all 1:50 dilution; Biolegend). Appropriate isotype controls (Biolegend) were used in all analysis. Then, PBMC were resuspended in 1% paraformaldehyde (PA; Sigma) and incubated for 30 minutes at 4°C. 6-hour cell cultures were resuspended in 1:10 permeabilization buffer (PERM-2; BD Biosciences), homogenized and incubated for 10 minutes at room temperature. After this period, PBMC were washed by centrifugation and resuspended in flow cytometry buffer (as previously described). Then, PBMC were stained with monoclonal antibodies for intracellular cytokines anti-IFN-γ PE-Cy7, anti-TNF Alexa700, anti-IL-10 PE and their respective isotype controls (Biolegend) for 30 minutes at 4°C. After other washes by centrifugation, PBMC were resuspended in 1% PA (Sigma). The cells were acquired on a FACSAria (with DIVA Software, BD Biosciences); 20,000 events/sample were acquired within the lymphocyte region for the *ex vivo*, and 50,000 events/sample for the 6-hour cultures. In flow cytometric analyzes, results are reported as % of median ± standard error of the median (SEM) for *ex vivo* data and range and quartiles (25^th^ and 75^th^ percentile) for data referring to cytokines-producing T cells.

### PAXgene whole blood RNA extraction and quantitative real time PCR (qRT-PCR)

Whole blood was obtained by venous puncture and stored in PAXgene tubes (BD Biosciences) at -80°C, for a period below six months. Whole RNA was prepared using PAXgene Blood RNA Kit (Qiagen) according to the manufacturer’s instructions. RNA was quantified on a Nanodrop ND-1000 spectrophotometer (Nanodrop, Wilmington, DE, USA). cDNA synthesis carried out using the Superscript III RT-PCR kit (Applied Biosystems, Branchbug, NJ, USA). *Taq*man real time PCRs were performed via the universal PCR Master Mix (x2) and specific primers and probes (Applied Biosystems). Briefly, PCR was performed in the ABI Prism 7000 sequence detection system (Applied Biosystems) at 50°C for 2 min, 95°C for 10 min, 45 cycles of 95°C for 15 s, and 60°C for 1 min. The studied genes were *TBX21* (Hs00203436), *GATA3* (Hs00203436), *RORC* (Hs01076122), *STAT3* (Hs00374280), *STAT4* (Hs00374280), *STAT6* (Hs00598625) and *FOXP3* (Hs01085834). Glyceraldehyde-3-phosphate dehydrogenase (*GAPDH;* 5’-CCGCATCTTCTTGTGCAGTG-3’) was used as an endogenous control and mRNA were quantified using the ΔCt method [ΔCt = Ct (target gene)—Ct (endogenous gene)]. qPCR conditions were the same as described above for the gene expression analysis (Applied Biosystems).

### Immunofluorescence studies

Punch skin biopsy (6 mm diameter) was obtained from BL patients at the onset of disease and/or before treatment of T1R leprosy. Skin specimens were embedded in Tissue-Tek O.C.T. Compound (Sakura Finetechnical, Tokyo, Japan), and snap frozen in liquid nitrogen. Longitudinal sections were cut at 16 μm on a cryostat (Leica CM 1850, Germany), mounted on gelatin-coated slides, and fixed in cold acetone for 15 minutes. Sections were washed 3 times with 10 mM PBS, pH 7.4, and then blocked by incubation with 10% normal goat serum (NGS, Invitrogen, USA) and 1% BSA (Bovine Serum Albumin, Sigma) for 1 h at room temperature. Incubation with the primary antibodies was performed overnight at 4°C in a humidity chamber, as such anti-CD4 (clone RPA-T4, isotype mouse IgG1), anti-CD8 (clone SK1, isotype mouse IgG1), anti-CD45RA (clone UCHL1, isotype mouse IgG2b) and anti-CLA (clone HECA-452, isotype rat IgM), all of them BD Pharmingen, were used in a 1:25 dilution. After wash with 10 mM PBS pH 7.4 (3 x 5 min each), sections were incubated at 90 min with rabbit anti-rat Alexa Fluor 488-conjugated and rabbit anti-mouse Alexa Fluor 633-conjugated, all obtained from Invitrogen and all at 1:200 dilution, in a humidity chamber, at room temperature. Secondary antibodies alone were used as negative controls. After a final series of washes in 10 mM PBS pH 7.4 (3 x 5 min each), cell nuclei were counterstained with DAPI (4’, 6’-diamidino-2-phenylindole, Sigma-Aldrich), and each coverslip was placed upside down on a slide containing a drop of SlowFade Antifade solution (Molecular Probes, OR, USA). The immunofluorescence analysis was performed with an Axio Observer Z1 Colibri microscope (Zeiss, Göttingen, Germany). For quantitative analysis of CD4+, CD8+ and CD45+ cells either expressing or not CLA, 10 microscopic fields were imaged and the number of positive cells was counted in each field. The results were obtained through a mean of fields’ counts, as determined by two independent observers. Images were processed via AxioVision software (Zeiss).

### Statistical and graphic analysis

The data were analyzed using GraphPad Prism version 6.0 (GraphPad, San Diego, CA, USA). The nonparametric Kruskal-Wallis test with post-test Dunns were employed to determine differences between stimulated (ML or SEB) and unstimulated cells (UNS). Mann–Whitney test was used to group comparisons, and Pearson’s test for a correlation analysis. We used a statistical significance level of *p<*0.05.

## Results

### Patients’ demographic and clinical features

According to Ridley and Jopling criteria [18], all leprosy patients examined in this study were classified by two experienced pathologists as borderline lepromatous (BL) or T1R. The mean age did not present a significant variation, as well as the gender of the studied individuals. T1R group showed a mean age of 41.25 years-old (ranging from 15 to 69 years-old), while BL group presented a mean age of 44.3 years-old (ranging from 18 to 63 years-old). Healthy volunteers (HV) showed a mean age of 38.3 (ranging from 20 to 59 years-old). As to T1R, 41.7% (n = 5) already presented the episode at the diagnosis of the disease and not treated until then, while 58.3% (n = 7) presented T1R during MDT. In T1R group, the mean of baciloscopic index (BI) corresponded to 2.27 (ranging from 1.25 to 3.75) while among non-reactional BL patients this mean corresponded to 3.28 (ranging from 2.5 to 4). In relation to the lepromin skin test (LST), all BL patients showed negative results, while only 25% (n = 3) of T1R patients were positive (≥ 5.0mm). As regards disability grade (DG), 83.3% (n = 10) of T1R patients already showed a loss of sensitivity or disabilities resulting from the disease, while among BL 60% (n = 6) already presented some kind of impairment (Table 1).

**Table 1:** Identification of study populations.

ID^a^	Form of leprosy	Gender	Age (ys.)	BI^b^	LST^c^	DG^d^	MDT^e^ (number of doses)
HR001	T1R	F	69	1.75	NEG	1	10°
HR002	T1R	F	25	2.5	NEG	1	8°
HR003	T1R	M	28	1.75	NEG	2	8°
HR004	T1R	M	22	2.5	POS	0	9°
HR005	T1R	F	29	1.5	NEG	1	NT^f^
HR006	T1R	M	54	3.75	NEG	3	NT
HR007	T1R	F	68	2.5	POS	2	NT
HR008	T1R	M	59	2.5	NEG	2	7°
HR009	T1R	F	42	1.5	NEG	1	NT
HR010	T1R	F	28	2.25	NEG	0	7°
HR011	T1R	F	56	3.5	NEG	3	NT
HR012	T1R	M	15	1.25	POS	1	9°
BS001	BL	M	29	3.75	NEG	2	NT
BS002	BL	M	61	4.0	NEG	2	NT
BS003	BL	F	63	3.75	NEG	1	NT
BS004	BL	F	56	2.75	NEG	1	NT
BS005	BL	M	31	2.5	NEG	0	NT
BS006	BL	M	33	3.0	NEG	0	NT
BS007	BL	F	42	2.55	NEG	1	NT
BS008	BL	F	53	3.75	NEG	0	NT
BS009	BL	M	57	4.0	NEG	1	NT
BS010	BL	F	18	2.75	NEG	0	NT
SD001	HV	F	25	-	-	-	-
SD002	HV	F	52	-	-	-	-
SD003	HV	F	29	-	-	-	-
SD004	HV	F	39	-	-	-	-
SD005	HV	F	42	-	-	-	-
SD006	HV	M	20	-	-	-	-
SD007	HV	M	32	-	-	-	-
SD008	HV	M	39	-	-	-	-
SD009	HV	M	59	-	-	-	-
SD010	HV	M	46	-	-	-	-

^a^ID: randomized code for each patient or heathy subject in order to safeguard their identity.

^b^BI: bacteriological index.

^c^LST: lepromin skin test [NEG = negative (5.0 mm) and POS = positive (≥5.0 mm)].

^d^DG: disability grade.

^e^MDT: multidrug therapy currently recognized by WHO (12 doses).

^f^NT: not treated.

### Determination of *ex vivo* T lymphocytes subsets

**Fig 1:**
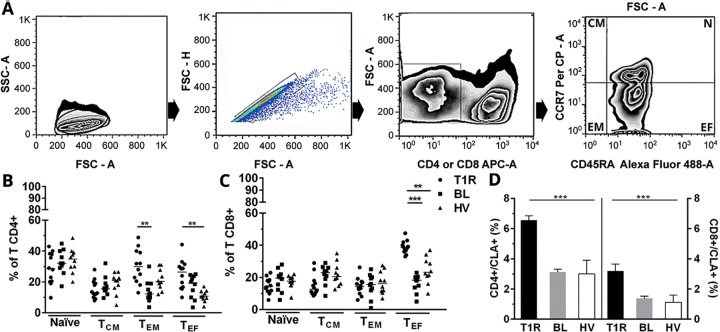
Increase of *ex vivo* CD4+ and CD8+ T subsets from BL patients at T1R. (A) Methodology for analysis of lymphocytic subsets isolated from newly obtained peripheral blood (time zero, T0) in FACSAria flow cytometer. Exclusion of dead cells (kit Live/Dead) and determination of lymphocyte region (FSC-A versus SSC-A; first dot plot at left). Exclusion of cell clumps by FSC-H X FSC-A (second dot plot at left). Determination of CD3+/CD4+ or CD3+/CD8+ T cells regions, by using specific monoclonal antibodies and isotype control (third dot plot) and subset analysis by marking with anti-CD45RA Alexa Fluor 488 versus anti-CCR7 PerCP antibodies (first dot plot at right). *Ex vivo* frequencies of CD4+ (B) and CD8+ T subsets (C). Significantly increased *ex vivo* frequency of double positive CD4+/CLA+ and CD8+/CLA+ T cells in T1R patients (D). Results represent a median ± SEM of isolated experiments from T1R (n = 12), BL (n = 10) and HV (n = 10). Significance levels are shown by the graphs, being **p<0.01 and ***p<0.001. Mann–Whitney test; comparison between groups.

CD3+/CD4+ and CD3+/CD8+ blood T cells of BL patients with and without T1R were analyzed immediately after PBMC isolation, as well as of healthy volunteers, to characterize the predominant *ex vivo* T cell populations in the two groups, following to the analysis methodology shown in Fig 1A. According to our results, CD4+ T cell subsets presented significant alterations in T_EM_ and T_EF_ cells from the studied groups (*p<*0.01; Fig 1B). As to CD8^+^ T cells, except for the T_EF_ cells (T1R *versus* BL *p<*0.001 and T1R *versus* HV *p<*0.01), other subsets showed no significant difference between groups (Fig 1C). Effector CD8+ T cell subsets from BL patients appear to be significantly increased at the onset of T1R, suggesting the participation of these cells at this reactional episode. To determine whether T1R alters the expression of Cutaneous Leucocyte-associated Antigen (CLA) molecules in circulating lymphocytes, CLA was evaluated *ex vivo* in CD4+ and CD8+ T cell subpopulations. In T1R patients, the CLA+ phenotype was significantly higher among T CD4+ than CD8+ T cells, but both showed significant differences when compared to BL and healthy volunteers (T1R versus BL and HV *p<*0.001; Fig 1D).

### *In vitro* analysis of T-cell memory subsets in response to *M*. *leprae*

**Fig 2:**
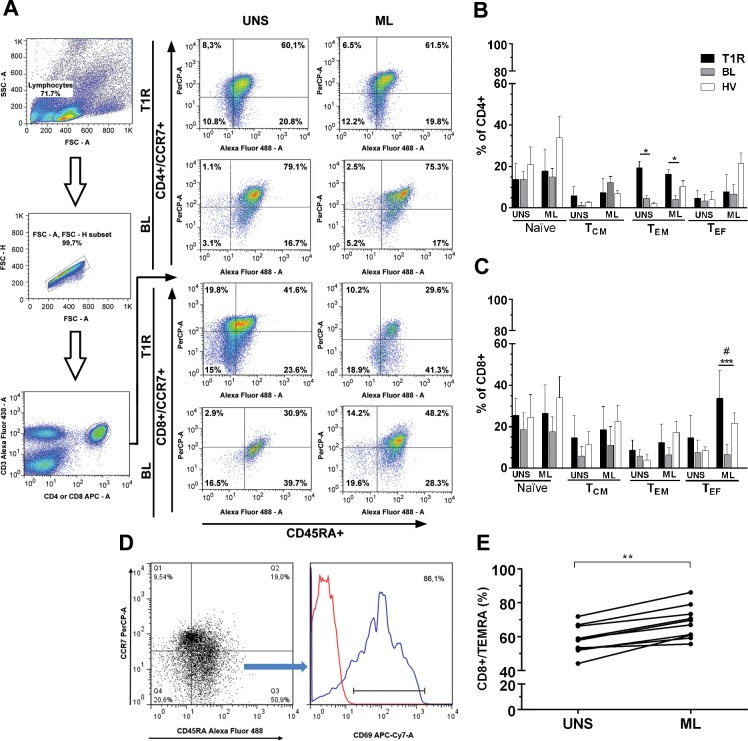
*M*. *leprae* increases the percentage of activated CD4+ (T_EM_) and CD8+ (T_EF_ and TEMRA) phenotypes in T1R patients. (A) Analysis methodology, as described by Fig 1 legend, in addition to subsets distribution upon unstimulated 6h-culture (UNS) and with 20μg/mL *M*. *leprae* (ML). The bars represent medians of CD4+ (B) and CD8+ (C) T activated cells. The lines above the bars represent SEM of isolated experiments from R1T (n = 12), BL (n = 10) and HV (n = 10). Kruskal-Wallis test with post-test Dunns were employed to determine differences between stimulated with ML or SEB (data not shown) and unstimulated cells (UNS). Mann–Whitney test was used for comparison between groups. Significance levels are shown by the graphs, being *p<0.05, **p<0.01 and ***p<0.001. # indicates CD69+^hi^ expressing T_EF_ cells (%), as shown by figure D. (E) Among T1R patients, CD8+/TEMRA cells were increased by *M*. *leprae* (ML) in comparison with UNS.

Then, in order to characterize the main subsets involved in the *M*. *leprae*-specific response shown by BL patients at the onset of T1R, we followed the cell marking technique mentioned in our methodology, as well as the standard analysis (Fig 2A). Upon comparison between the groups, the activation of CD4+ T cells by *M*. *leprae* did not undergo significant changes, except in T_EM_ subsets. In this subset, the difference was significant among T1R and BL groups and also in T1R patients regardless of the culture conditions (UNS and ML, *p<*0.05; Fig 2B). Among CD8+ T cells, all subsets presented an increased activation threshold, even in unstimulated cultures. However, such values did not show significant differences in comparison with the two other groups evaluated under the same conditions. *M*. *leprae* induced a significant increase of CD8+ T_EF_ in T1R group when compared to BL patients, (*p<*0.001), as well as to control group (*p<*0.05; Fig 2C). During the experiments, cultures were also stimulated with SEB (as positive control) and, as expected, all the results were positive (data not shown). The predominance of previously activated CD8+ T lymphocytes in T1R group, as well as after *M*. *leprae* stimulation, allows us to suggest that these subsets may contribute to the appearance of T1R. In addition to this finding, *M*. *leprae* also promoted an increase of CD8+/CD45RA+/CCR7-/CD69+^high^ cells (Fig 2D), as known as terminally differentiated effector T cells (TEMRA) in T1R patients (*p<*0.05; Fig 2E). Finally, we note a negative correlation between *M*. *leprae*-specific CD8+ T_EF_ cells, particularly between IFN-γ producing one, and the positivity to LST at the onset of T1R (r = -0.187, p = 0.47). These results are shown in S1 Fig.

### Frequency of IFN-γ-, TNF- and IL-10-producing T cell subsets in response to *M*. *leprae*

**Fig 3:**
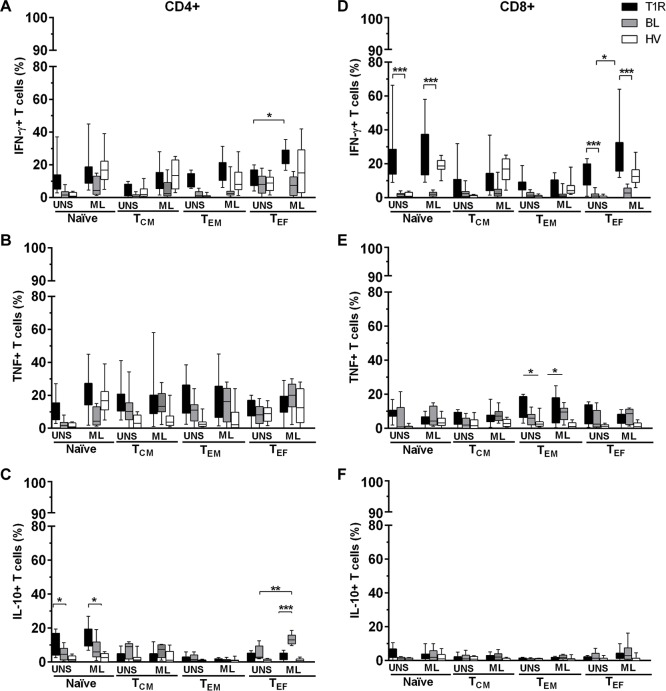
IFN-γ- and TNF-producing CD4+ and CD8+ T cells are predominant in T1R group, while IL-10-producing CD4+ T cells are increased in non-reactional BL patients. IFN-γ-, TNF- and IL-10-producing CD4+ (A-C) and CD8+ (D-F) T subsets in 6h-PBMC culture were: unstimulated (UNS), 20μg/mL *M*. *leprae* (ML) and 1μg/mL SEB (data not shown). Results are reported as % of median and interquartiles (25^th^ and 75^th^ percentile) in T1R (n = 12), BL (n = 10) and HV (n = 10) groups. Kruskal-Wallis with post-test Dunns were employed to determine differences between stimulated (ML or SEB) and unstimulated cells (UNS). Mann–Whitney test was used to group comparisons; *p<0.05, **p<0.01 and ***p<0,001.

IFN-γ, TNF and IL-10 are mediators traditionally associated with leprosy pathogenesis *per se*. In order to evaluate the participation of these cytokine-producing T lymphocytes in T1R, both *M*. *leprae* -stimulated and unstimulated PBMC cultures were permeabilized, stained and analyzed by flow cytometry. We observed a general increase in the frequency of all IFN-γ-producing CD4+ T subsets from T1R group, regardless of culture conditions. However, CD4+/IFN-γ+ T_EF_ in T1R group presented a significant difference between UNS- and ML-stimulated cells (*p<*0.05; Fig 3A). TNF-producing CD4+ T cells also presented a higher frequency in T1R group, both in unstimulated cultures and in response to *M*. *leprae*. However, there was no significant difference in relation to BL patients or to healthy volunteers. IL-10 producing CD4^+^ T_NAÏVE_ were significantly increased in T1R group, in comparison with BL patients and with healthy volunteers. This finding was observed both in UNS- (*p<*0.05) and in ML-stimulated cultures (*p<*0.05; Fig 3C). IL-10+/CD4+ T_CM_ and T_EM_ did not changed under any culture conditions. *M*. *leprae* induced an increased frequency of CD4+/IL-10+ T_EF_ in BL group (*p<*0.05; Fig 3C). As observed among CD4+ T cells, the expression of IFN-γ-producing CD8+ T_NAÏVE_ also appeared to be significantly increased in T1R group in comparison with BL and HV subjects, under both culture conditions (*p<*0.05; Fig 3D). CD8+/IFN-γ+ T_CM_ and T_EM_ frequencies were also increased in T1R group in relation to BL patients and HV individuals. However, only IFN-γ-producing CD8+ T_EF_ cells presented a significant difference between culture conditions and among the studied groups (*p<*0.05; Fig 3D). The frequency of TNF-producing CD4+ T subsets did not differ between studied groups. However, we observed a significant increase in TNF+/CD8+ T_EM_ cells (*p<*0.05; Fig 3E). Finally, CD8+/IL-10+ T subsets also did not differ. As expected, SEB increased the frequency of all the studied cytokine-producing T cell subsets (Fig 3A–3F). As combined with prior data, these results suggest that, at the onset of the reactional episode, IFN-γ-producing T cells may have influenced the increased TEMRA phenotype expression found in T1R group. Among BL patients, the increased frequency of IL-10+ CD4+ T subsets may have exert a suppressive role by inducing an inhibitory effect on cell-mediated immune responses towards *M*. *leprae*.

### Gene expression of transcription factors involved in T lymphocytes differentiation

**Fig 4:**
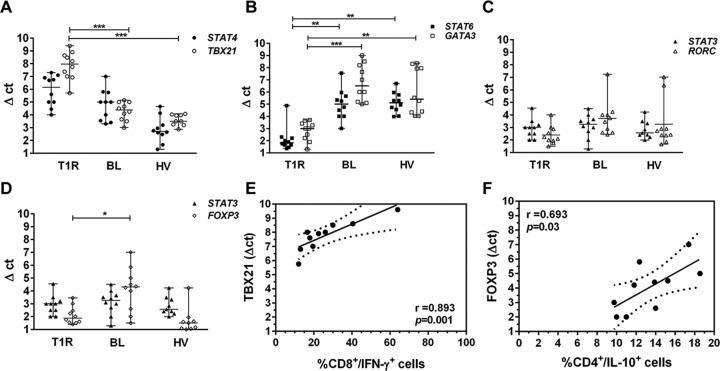
Increased *TBX21* in T1R group is associated with IFN-γ+-producing CD8+ T cells, while *FOXP3* is associated with IL-10+-producing CD4+ T cells in BL patients. Scattergram showing the expression of gene pairs *STAT4/TBX21* (A), *STAT6/GATA3* (B) *STAT3/RORC* (C) and *STAT3/FOXP3* (D). Quantitative RT-PCR analysis in peripheral blood of T1R patients (open symbols) and BL (closed symbols) and HV (open triangles). The y-axis of each graph represents the relative expression of the respective genes calculated using the Δct method and normalized against *GAPDH* mRNA. Results are reported by Δct mean ± range of 10 independent samples of each group. *p<0.05, **p<0.01 and **p<0.001 (Mann–Whitney test). Spearman rank correlations of *TBX21* expression with IFN-**γ**-producing CD8+ T cells from T1R group (E), and *FOXP3* expression with IL-10-producing CD4+ T cells from BL patients (F) are shown.

In order to evaluate the gene expression of transcription factors responsible for T lymphocytes differentiation, we carried out a real time qPCR with cDNA collected from peripheral blood obtained by PAXGene tube. We used *STAT4/TBX21* gene pair to identify Th1, *STAT6/GATA3* for Th2, *STAT3/RORC* for Th17 and *STAT3/FOXP3* for Treg profile. mRNA expression of *STAT4/TBX21* genes was increased in T1R group, in comparison with the same number of BL patients, as well as healthy volunteers (HV group). In fact, *TBX21 per se* was significantly higher among T1R patients (*p<*0.001; Fig 4A). Then, we evaluated *STAT6/GATA3* expression, which was higher among BL patients (*p<*0.001) and HV group (*p<*0.05) than in T1R (Fig 4B), thus suggesting a tendency to the development of Th2 profile in BL and HV. As we observed the genes inducing a differentiation for Th17 profile, we noted a slight increase of *RORC* and *STAT3* among non-reactional BL patients in comparison with T1R group, without significant difference (Fig 4C). The last transcription factor evaluated was *FOXP3* expression, that presented a difference between the groups, as the values were significantly higher in BL patients than in T1R group (*p<*0.05; Fig 4D). As described above, *STAT3* expression did not allow a discrimination between BL patients either affected or not by T1R. Likewise, it was not observed among healthy volunteers. Our gene expression analysis suggests T lymphocytes differentiation in T1R refers to Th1 phenotype, with an increased expression of *TBX21*, while, in non-reactional BL form, it relates to Th2 through an increase of *STAT6* and *GATA3*. One should not rule out the participation of Treg among non-reactional BL patients, as *FOXP3* expression was significantly increased in this group, in comparison with T1R patients (*p<*0.05; Fig 4D). As IFN-γ producing CD8+ T_EF_ cells and *TBX21* were significantly higher in T1R group, we considered a possible correlation between them. Fig 4E discloses a strong correlation obtained from such analysis (r = 0.8936, *p<*0.001). Likewise, we observed a significant positive correlation between IL-10-producing CD4+ T_EF_ and *FOXP3* in BL group (r = 0.693, *p<*0.05; Fig 4F). We still carried out a correlation analysis between gene expression of all the evaluated transcription factors and all studied cytokine-producing T lymphocytes subsets in T1R and Bl groups, but reached no significant results (data not shown).

### Measurement of T cell subsets and homing regulation marker in skin lesions by immunofluorescense

**Fig 5:**
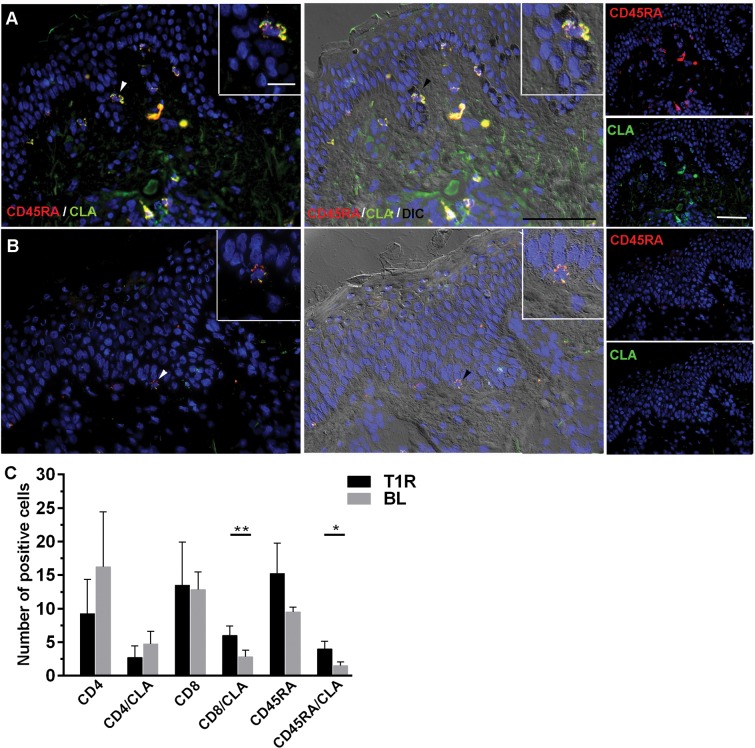
CD8+/CLA+ and CD45RA+/CLA+ T lymphocytes are predominantly expressed in T1R lesions. Immunofluorescence assays were performed to determine the number of T cell markers (CD4, CD8 and CD45RA) expressing or not CLA. Representative images presented CD45RA+ (*red*, Alexa Fluor 633; upper right panels) and CLA+ (*green*, Alexa Fluor 488; lower right panels), colocalization (*yellow*, white arrow head and insert; left panels). The nuclei (*blue*) were stained using DAPI. Scale bar = 50 mm. Data are representative of 3 specimens of each group, being one T1R (A) and one BL patient (B). Images were visualized and obtained by a Zeiss Colibri fluorescent microscope. The graph shows single and double-positive CD4+/CLA+, CD8+/CLA+ and CD45RA+/CLA+ cells in T1R and BL skin infiltrates (C). *p<0.05 and **p<0.01 (Mann-Whitney test; group comparisons).

CLA (Cutaneous Leucocyte-associated Antigen), member of the selectin family, is expressed in T cells that can be driven to inflamed tissues through endothelial cells. In order to examine any possible regulation of CLA on T cell subsets in the context of T1R, we used immunofluorescence to analyze skin lesions in biopsies obtained from 6 studied patients (3 T1R and 3 BL). We observed a preferential distribution of colocalizated CD45RA+/CLA+ cells through the intraepithelial region of T1R lesions. Fig 5A shows a representative experiment of T1R studied patient. However, in non-reactional BL patients these cells were rare and CLA+ staining intensity was smaller (Fig 5B). With respect to the number of T cells in the lesions, as observed in Fig 5C, CD4+ T cells either expressing or not CLA were found in a higher number in lesions from BL patients compared with T1R group, although there was no significant difference. However, CD8+ cells were detected in a higher number in reactional lesions, as well as CD8+/CLA+ T cells, which appeared to be more significantly present in these inflammatory infiltrates (*p<*0.01). Likewise, in terms of maturation profile, CD45RA+ cells were found in a higher number in lesions from T1R patients. Finally, in relation to CD45RA+/CLA+ cells, the difference between the groups was significant (*p<*0.05). According to the cell distribution in the lesions, it is possible that double-positive cells (CD45RA+/CLA+) detected in a higher number in T1R group are CD8+ T cells, being thus compatible with TEMRA phenotype as observed in the blood from such patients. A relevant limitation for the demonstration is this hypothesis lies on the impossibility of evaluating CCR7 marker in skin lesions.

## Discussion

In spite of the significant reduction of leprosy *per se* worldwide since MDT implementation, reactional states still remain a major public health concern. A reactivation of cellular immune response in borderline leprosy patients during T1R was previously described, although most studies do not discriminate borderline forms [7, 12, 13]. *M*. *leprae*-specific hyporesponsivity in blood leukocytes from classical BL patients is well known [10]. Nevertheless, as T1R patients show granulomatous skin lesions, one should not rule out the participation of T lymphocytes in the immunopathogenesis of such episodes [14]. Thus, considering that these individuals present different clinical and immunological features, this work only focused on BL patients who developed Type 1 Reaction (T1R), by comparing them with newly diagnosed patients (without reactional signs and symptoms) before MDT, as well as with healthy volunteers (HV).

In our study, T1R group was mostly composed by female individuals, showed a mean age below the non-reactional group. These data are in perfect accordance with works from other groups on risk factors to the onset of reactions [4, 5]. Of note, our T1R group also presents a high rate (83.3%) of patients with some constant level of disability, such as nerve impairment and physical disabilities. In relation to lepromin (LST), all non-reactional BL patients showed negative skin test, thus indicating hyporesponsiveness or anergy, while 25% of T1R patients were responsive, suggesting that both T1R and some BL individuals were more responsive to *M*. *leprae* antigens in the skin. Indeed, leprosy reactions may induce a strong release of antigens from *M*. *leprae* fragments deriving from the use of antimicrobial drugs. Nevertheless, we did not observe significant clinical and/laboratorial differences between patients who came to present T1R, in comparison with patients in treatment.

We analyzed PBMC cells phenotype just upon T1R diagnosis. The significant *ex vivo* increased frequency of CD4+ T_EM_/T_EF_ and CD8+ T_EF_ cells found in T1R group suggested an intravascular leukocyte activation at the onset of reactions. When compared to naïve or central memory T cells, these subsets, which are great IFN-**γ**producers, were already shown to be able to mount faster responses [19]. As still refers to findings from *ex vivo* PBMC, the significant increase of CLA+ cells both in CD4+ and CD8+ T cells in T1R group, in contrast to non-reactional BL group, corroborates the hypothesis that, in T1R, part of intravascular T cells may migrate to skin and develop local inflammatory response. Austin *et al*. showed that T cells directed to the skin via CLA may also mediate inflammatory responses and CLA+ T cells have previously been shown to be enriched in the inflammatory lesions of psoriasis where Th1 cytokine producing cells are thought to have a pathological role [20].

Therefore, we also aimed at the identification of *M*. *leprae*-specific T subsets in T1R patients. According to *in vitro* analysis, *M*. *leprae* induced a significantly increased frequency of activated CD4+ T_EM_ and T_EF_, as well as of CD8+ T_EF_ cells in this group. The venous blood collection from T1R individuals just upon appearance of early reactional signs and symptoms corroborates our findings concerning the activation of T cells still in blood. Prior studies indicate a participation of effector and effector memory T cells in the immunopathology of severe cases of both human pulmonary tuberculosis and cutaneous leishmaniosis, which are also caused by intracellular pathogens [21, 22]. Moreover, we observed that *M*. *leprae* induced a significantly increased CD69 expression in CD8+/CD45RA+/CCR7- T cells, and indicated terminally differentiated effector T cells (TEMRA) in T1R group. The high frequency of *M*. *leprae*-specific CD8+ TEMRA cells may be associated with T1R severity, as the two patients showing the highest CD69 expression (>85% of positive cells) also presented the most intense inflammatory response (HR006 and HR011 patients; data not shown). Similar findings were disclosed by Oliveira *et al*., who also demonstrated an increase in both CD4+ and CD8+ TEMRA cells in peripheral blood leucocytes in co-infected HIV/leprosy patients showing T1R. Authors also suggested that CD8+ T_EM_ cells triggered T1R in HIV/leprosy patients [23].

To understand further the functional activity of T lymphocytes subsets, we characterized the frequency of IFN-γ, TNF and IL-10-producing T cells. The first two cytokines are known to be important in leprosy immune reactivation [8, 24], [25], while IL-10 is correlated with the pathogenesis of multibacillary forms [26, 27]. In T1R group, even without stimuli, we observed a higher frequency of IFN-γ-producing CD4+ T_EF_ in comparison with BL and HV groups. *M*. *leprae* induced a significantly increased frequency of IFN-γ-producing CD4+ T_EF_. Likewise, the frequency of unstimulated and antigen-specific naïve and effector IFN-γ-producing CD8+ T cells was higher in T1R group. Such increased frequency of IFN-γ-producing T cells among T1R patients was consistent with prior studies, although such works were performed with skin lesions or assessed cytokine production in serum or in PBMC culture supernatants [7, 28].

Although more frequent in ENL, previous studies indicated that TNF mediates immune-pathologic effects, such as fever and tissue damage, in both type 1 and type 2 leprosy reactions [29, 30]. Our work did not find a significant increase of TNF-producing CD4+ T subsets, upon comparison of both culture conditions and studied groups. However, the frequency of TNF-producing CD8+ T_EM_ cells was significantly increased in T1R patients in comparison with BL and HV groups. This finding occurred both in unstimulated cultures and in response to *M*. *leprae*. At the onset of reactions, T1R CD8+ T_EM_ cells are possibly activated by *M*. *leprae* components, and may produce TNF and play a cytotoxic role. In another possibility not investigated in this study, such TNF-producing cells may be unconventional double-positive CD8/γδ T cells. A previous work from our group demonstrated an increased expression ofγδ+ T cells in lepromatous patients with severe type 2 reaction and increased TNF level in blood circulation [31].

Moreover, T1R group presented a significantly increased frequency of *M*. *leprae*-specific CD4+/IL-10+ T_NAÏVE_ cells. Considering the marked IL-10 action on downregulation of inflammatory responses, through inhibition of Th1 responses, such result was surprising. IL-10 is produced by several T lymphocytes subsets, and is not subject to an epigenetic regulation, as is the case with IFN-γ [32]. Possibly, these IL-10-producing T cells could counterbalance the activation of IFN-γ- and TNF-producing-T cells in T1R. As to reactional group, the significantly increased frequency of *M*. *leprae*–induced CD4+/IL-10+ T_EF_ is compatible with findings from other works that showed more abundant IL-10 levels produced by type 2 macrophages during mycobacterial infections, thus resulting in a decreased nitric oxide production and in enhanced intracellular bacterial growth [33, 34]. A recent family-based meta-analysis study actually confirmed an association between IL-10 promoter polymorphisms and leprosy. However, the authors did not study patients presenting reactional episodes [35].

As to cytokine participation in the immunoinflammatory response observed in T1R group, the increased frequency of IFN-γ-producing T cells may have influenced the higher expression of both effector and memory phenotypes found in the blood from these patients. Within this context, Obar et al., demonstrated that higher inflammatory levels and increased antigen-specific responses favored the generation of short-term CD8+ T_EF_ cells [36]. On their turn, the maintenance of T_EM_ requires ongoing antigen stimuli, and it is possible that, in T1R, intense fragmentation/death of *M*. *leprae* leads to the release of new epitopes resulting in enhanced antigen presentation, which activate such cells. Thus, our data shed light on how varying the context of T cell priming alters downstream effector and TEMRA CD8+ T cell differentiation in leprosy type 1 reaction.

Gene expression analysis of transcription factors for T lymphocytes differentiation showed a significant increase of *TBX21* expression in T1R patients, in comparison with BL group. As we compared the two groups, *STAT4* was also slightly increased, although this difference was not significant. *TBX21*, also designed *T-bet*, was proposed to be the master switch for Th1 development based on its IFN-γ induction and its direct activation of IFN-γ reporter activity [37, 38]. In leprosy, Quiroga *et al*. demonstrated by western blot that *TBX21* is expressed in PBMC from BT patients, and correlated this finding with IFN-γ production in supernatants from *M*. *leprae-*stimulated cultures [38]. Our data suggest that the pro-inflammatory microenvironment found in T1R may favor an increased *TBX21* expression. In contrast, BL patients displayed a significant increase of transcriptional factors pair of genes that had driven a Th2 differentiation (*STAT6* and *GATA3*). It was already suggested that *TBX21*:*GATA3* ratio may reflect Th1/Th2 cytokine balance, and that the lower is such ratio, the higher will be the differentiation for Th2 profile [39]. As expected, our study showed that this ratio was considerably reduced in BL group. Among these patients, we noted a significant increase of *FOXP3*, which is compatible with Th1 pattern inhibition. Both natural and induced Treg cells were observed in skin lesions and peripheral blood in lepromatous leprosy and the immune suppression observed during the course of the disease was linked to an increased *FOXP3* expression [40]. In our work, we noted a strong correlation between *TBX21* expression and the frequency of IFN-γ-producing CD8+ T cells, which corroborates our hypothesis as to the T cell activation in T1R group. The strong negative correlation between *FOXP3* expression and IL-10-producing CD4+ T cells at the onset of T1R in our patients indicates a possible inhibition of Treg cells in T1R. Consistent with our observation, Geluk *et al*. showed a decrease in Treg subsets in a recent case report focusing a BL patient at the onset of T1R [41]. More recently, a work showed increased Th17 and reduced Treg cells among T1R patients. The authors associated such findings with an increased IL-17A gene expression, IL-17 and IL-6 levels, and with reduced gene expression of *FOXP3* and accompanied by decrease of TGF-βproduction. Nevertheless, the studied T1R patients presented a BT form, which is known to differ from BL patients under immunological aspects [42]. Among non-reactional BL patients, we found a positive correlation between *FOXP3* expression and IL-10-producing CD4+ T cells. In parallel, other authors demonstrated an increased CD4+/IL10+ Treg cell number in PBMC of lepromatous (BL/LL) patients and increased IL-10 levels in culture supernatants [43]. So far, our results allow us to hypothesize that T1R in BL patients is triggered by an early *M*. *leprae* activation of circulating effector and memory T cells. These cells may rapidly migrate towards skin lesions and nerve trunks, thus leading to the appearance of reactional symptoms.

A prior study from our group detected increased macrophages, epithelioid cells and dendritic cells in skin lesions from BL patients at T1R. Authors attributed such fact to an abrupt release of immune inflammatory mediators disrupting the predominantly immunosuppressive milieu in multibacillary leprosy [44]. In our work we found a significantly higher number of both double-positive CD8+/CLA+ T cells and CD45RA+/CLA+ in T1R skin biopsies. It is well known that CLA is expressed in T cells that can be driven to inflamed tissues, and that *M*. *leprae* specifically up-regulated CLA [45]. Of note, we observed a preferential distribution of co-localized cells through the intraepithelial sites of T1R lesions, bordering basal layer. These cells may have been possibly attracted to the skin by chemokines secreted by macrophages and/or keratinocytes. IFN-γ inducible protein CXCL10/IP-10 was demonstrated to be increased in T1R lesions [46]. In other study, immunohistochemistry analysis showed increased CCR5 (RANTES receptor) and CXCR2 levels in biopsy specimens from T1R patients [47]. Another possibility that still requires investigation from our group lies on the double-positive cells (CD45RA+/CLA+) detected in a higher number in T1R group, that may also be CD8+ T cells. Consistent with such a possibility, a recent work in cutaneous leishmaniosis demonstrated that both effector and memory T cells co-expressing CLA might potentially influence the cell composition of inflammatory infiltrate, thus contributing to the severity of the disease [48]. As this study was primarily focused on T1R patients, we understand that further deeper studies of skin lesions from BL patients before, during and after the reaction may clarify the nature of *in situ* interactions between potentially migrating and/or resident immune cells. In fact, skin lesions share similar features with peripheral blood mononuclear cells, and provide additional information on local immune responses that may be collaborating with T1R pathogenesis in BL leprosy.

## Conclusions

Our work provides evidences of a potential role played by circulating CD4+ T_EM_ and CD8+ T_EF_ and TEMRA lymphocytes and pro-inflammatory cytokines at the onset of T1R in BL patients. In these patients, immune cell activation inside the lesion sites, such as skin and peripheral nerves, appears to be directly influenced by circulation. However, neither correlation nor significant difference between reactivity to cutaneous test, treatment status, disability grade and *ex vivo* or *in vivo* results were observed. Given the complex interaction between effector/memory T cells and cytokines/chemokines, longitudinal follow-up studies may be required to the clarification of such mechanisms.

## Supporting Information

S1 Fig Reactivity to LST is not a marker of T1R in BL patients. Negative correlation between cutaneous test for leprosy prognostic and CD8+/IFN-γ+ T_EF_ cells frequency from T1R patients. Data from LST are shown in millimeters (mm) of cutaneous induration for a better visualization of results. Spearman correlation test.
(TIF)
