# Supplementary figures and images for: Role of TEFFECTOR/MEMORY Cells, TBX21 Gene Expression and T-Cell Homing Receptor on Type 1 Reaction in Borderline Lepromatous Leprosy Patients
Source: PLoS One. 2016 Oct 20;11(10):e0164543. doi: 10.1371/journal.pone.0164543 (PMC5072666; doi:10.1371/journal.pone.0164543)

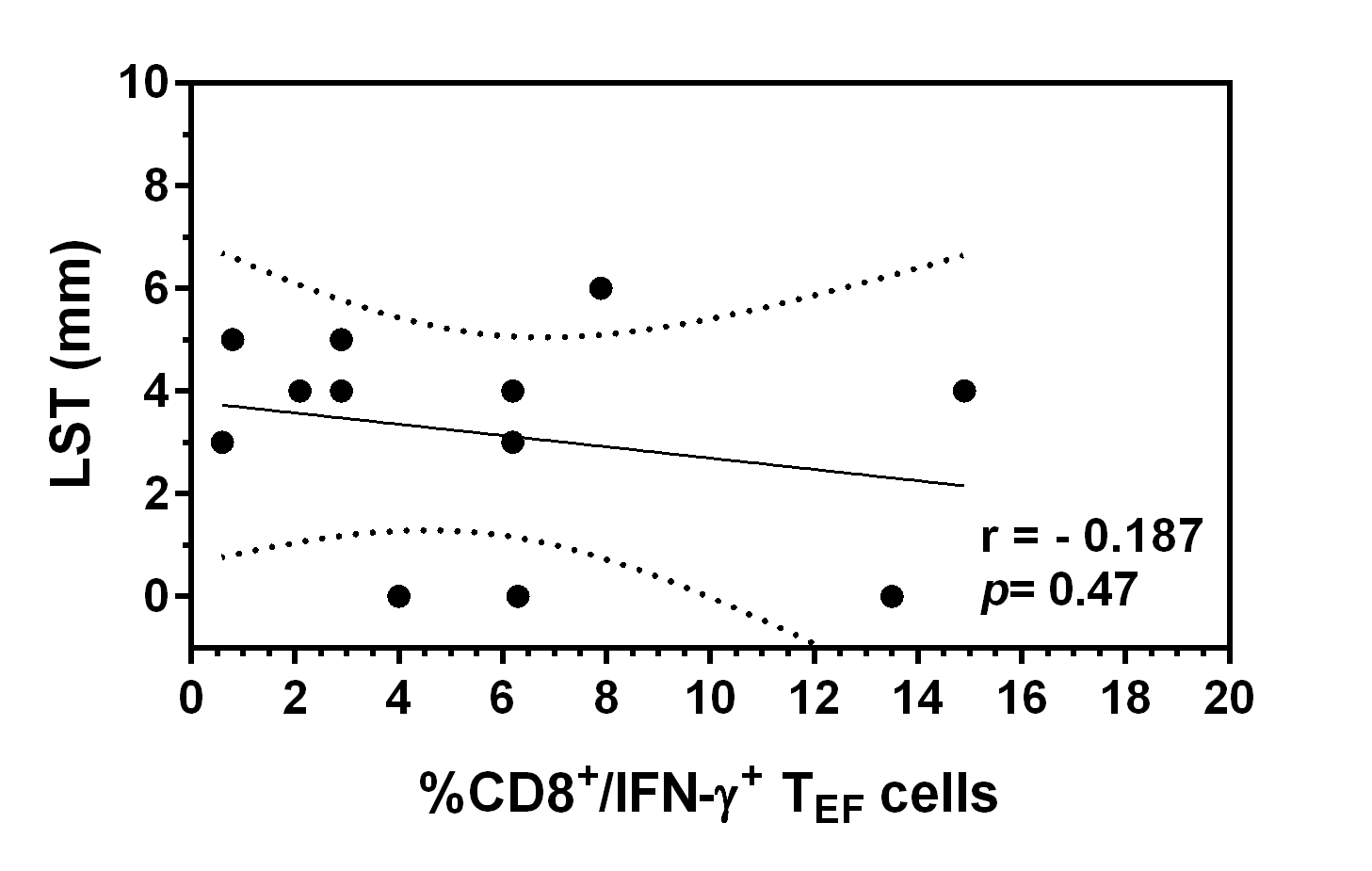

Supplement: S1 Fig — Negative correlation between cutaneous test for leprosy prognostic and CD8+/IFN-γ+ TEF cells frequency from T1R patients. Data from LST are shown in millimeters (mm) of cutaneous induration for a better visualization of results. Spearman correlation test. (TIF) [file pone.0164543.s001.tif]
